# Supplementary material for: Demixing fluorescence time traces transmitted by multimode fibers
Source: Nat Commun. 2024 Jul 26;15:6286. doi: 10.1038/s41467-024-50306-z (PMC11282286; doi:10.1038/s41467-024-50306-z)
Supplement: Supplementary file 1 — Supplementary Information [file 41467_2024_50306_MOESM1_ESM.pdf]

Demixing fluorescence time traces transmitted by multimode fibers

Caio Vaz Rimoli[1,2], Claudio Moretti[1], Fernando Soldevila[1], Enora Brémont[2], Cathie Ventalon[2*], Sylvain Gigan[1*]

[1] Laboratoire Kastler Brossel, ENS-Université PSL, CNRS, Sorbonne Université, Collège de France, 24 Rue Lhomond, Paris, F-75005, France.
[2] Institut de Biologie de l'ENS (IBENS), Département de biologie, École normale supérieure, CNRS, INSERM, Université PSL, 75005 Paris, France
* These authors jointly supervised the work

SUPPLEMENTARY INFORMATION

List of contents

- **Figure S1:** The proof-of-principle setup
- **Figure S2:** Residual error estimation (fidelity plot) for NMF with different factorization ranks
- **Figure S3:** Results of the proof of principle experiment presented in Figure 2, analyzed with an input rank for NMF of R = 4.
- **Figure S4:** Results of the proof of principle experiment presented in Figure 2, analyzed with an input rank for NMF of R = 7
- **Figure S5:** All the fingerprint results of the proof of principle experiment presented in Figure 2 (analyzed with NMF rank R = 9)
- **Figure S6:** NMF denoising effect on spatial fingerprints
- **Figure S7:** Neuropil experiment results
- **Figure S8:** Neuropil experiments estimated GT ranks: the fidelity plots
- **Figure S9:** The miniscope detection sensitivity to a single source
- **Figure S10:** Pattern shape dependence on symmetrically positioned beads (with miniscope)
- **Figure S11:** Testing the results for NMF when spatially binning the recorded video

- **Supplementary Note 1:** Estimating the number of fluorescence sources with NMF: the NMF rank study
- **Supplementary Note 2:** Comparing GT-NMF and GT-GT time trace correlation coefficients
- **Supplementary Note 3:** Scattering properties of Parafilm M®
- **Supplementary Note 4:** Number of available scattering fingerprints (sources) to be demixed

- **Supplementary Information References**

Figure S1: The proof-of-principle setup

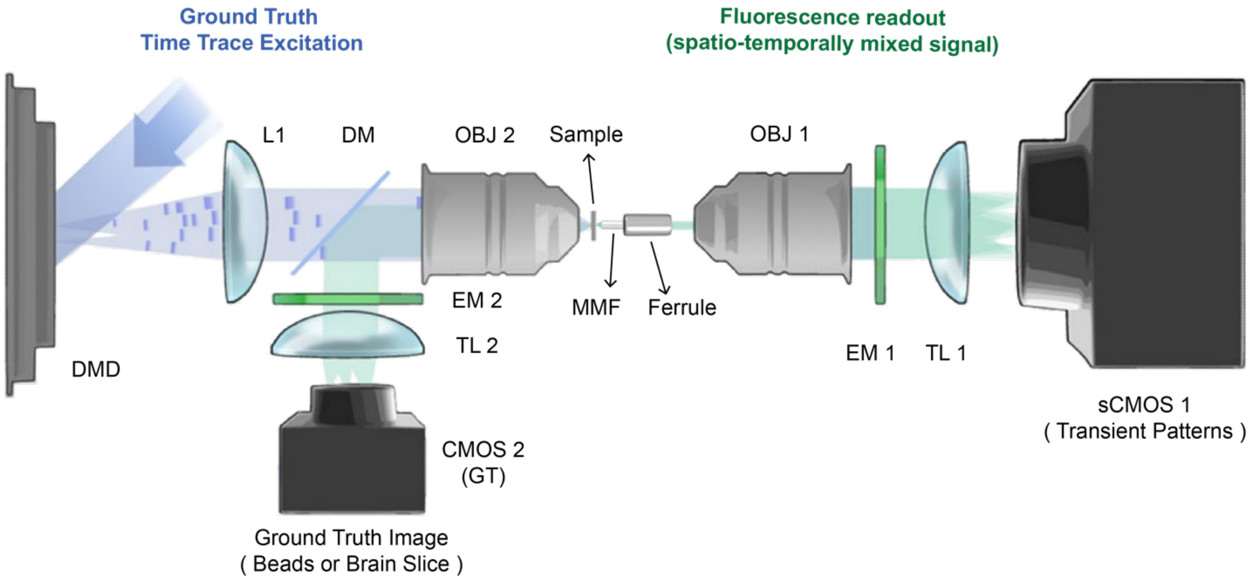

Figure S1 - The proof of principle optical setup. A 473 nm blue laser (LSR-0473-PFM-00100-01, Laserglow Technologies) illuminates a digital micromirror device surface (DMD from Texas Instruments: DLP LightCrafter 6500, same as [43]), that was used to create the ground truth (GT) excitation of each fluorescence source present in the sample plane (see sample details in the methods). A tube lens (L1, LA1708-A, Thorlabs) and the lower objective OBJ 2 (Plan-NEOFLUAR × 20, 0.5 NA, Zeiss) were used in the excitation path. For the ground truth excitation, genetically encoded calcium indicator (GECI) traces were obtained from neuronal recordings available in the literature, as we did before in previous experiments [43,57]. After excitation, the florescent signal propagated through the short multimode fiber (see fiber-ferrule preparation details in the methods) and the fluctuating fluorescent scattering fingerprints on the tip of the fiber were imaged onto a scientific complementary metal-oxide-semiconductor (sCMOS) camera (Iris 15 sCMOS, Teledyne Photometrics) by a microscope objective (OBJ 1, RMS10X PLAN ACHROMAT 0.25NA, Olympus) and a tube lens (TL 1). An emission bandpass filter (MF530-43, Thorlabs) was used in the detection path to block the blue excitation light. In addition, the system has a control path to image the fluorescence sample directly, so that it would be possible to obtain the ground truth image of the sample without passing through the fiber. This is done by imaging the backpropagating fluorescent light from the sample in reflection mode. For this path, a dichroic beam splitter (DM, FF496-SDi01, Semrock) was used to collect the fluorescent signal in reflection and the sample plane was imaged onto a CMOS camera (ACE2014-55um, Basler) after another tube lens (TL 2). We typically used a laser power of $3{,}0.10^{-8}$ W per 10µm diameter area (bead size), resulting in a local intensity of $3{,}8.10^{-2}$ W/cm². Synthetic fluorescence traces were generated like previous work from our team, using the spike activity dataset from real available datasets acquired in the mouse visual cortex, and converting them into calcium (excitation) traces (MLSpike from Vanzetta's group: Deneux, T., *et al.* Accurate spike estimation from noisy calcium signals for ultrafast three-dimensional imaging of large neuronal populations *in vivo*. *Nat Commun* 7, 12190 (2016). https://doi.org/10.1038/ncomms12190) using a GCaMP6s physiological model and resampled at 10 Hz. The analog calcium transient profiles were designed by changing the dwell time of the excitation in relation to a longer, but constant, detection window time - similar to previous works from our team. The DMD can control the excitation beam fast (>10kHz) concerning the detection window, allowing it to mimic an analog time trace profile very smoothly in the video recording. A higher (resp. lower) fluorescence intensity from a given bead corresponds to a longer (shorter) excitation dwell time during a given detection window. The detection window in the experiments were: 500 ms (Figure 2 - the 6-bead experiment, camera dynamic range of ~10.000), 1000 ms (Figure 3 - the 26 bead embedded in 50 µm thick agarose layer sandwiched between coverslips, camera dynamic range of ~1.500), 2000 ms (Figure 4 - the 26 bead embedded agarose layer sandwiched between coverslips + single Parafilm M® layer on top, camera dynamic range of ~1.500), 500 ms (Figure 5 – the 21 bead experiment with 11 neuropil sources, camera dynamic range of ~6.000), 4000 ms (Figure 6 – 4 neurons in 50 µm brain tissue slice of the Gad-EGFP labeled neurons from the cortex, camera dynamic range of ~450). Therefore, most of the experiments used a moderate dynamic range compared to the available 16-bit. Figure adapted from Moretti, C. & Gigan, S. *Nat Photonics* **14**, 361–364 (2020).

Figure S2: Residual error estimation (fidelity plot) for NMF with different factorization ranks

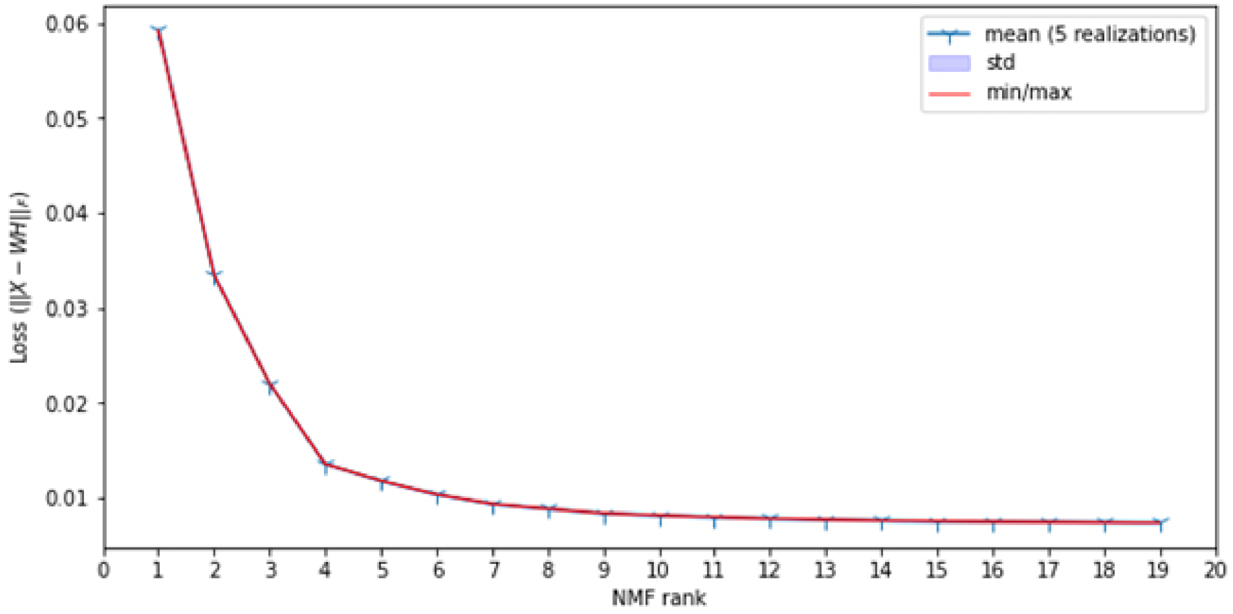

Figure S2 – Residual error estimation (fidelity plot) for NMF with different factorization ranks. The sample used here is the same as in Figure 2 and contains 6 beads. One might see that the fidelity curve changes its curvature when the tested rank values gets closer to the GT expected rank (6 beads + 1 background). After that, the NMF performance slowly reaches a plateau. For large NMF rank values, the extra fingerprints and time traces obtained are replicas of the GT signal. NMF model parameters were the same as methods, only changing the rank value. Mean value and standard deviations (std) over 5 realizations with 3000 interactions.

Figure S3: Results of the proof of principle experiment presented in Figure 2, analyzed with an input rank for NMF of R = 4.

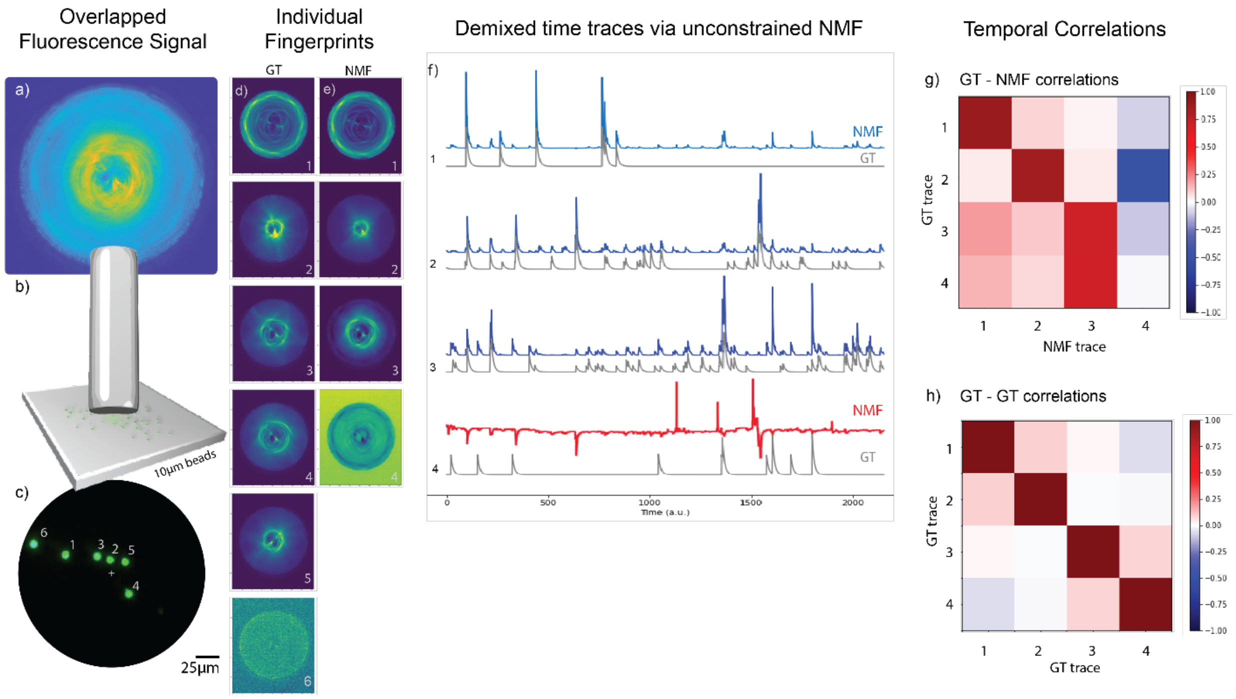

Figure S3 – Results of the proof of principle experiment presented in Figure 2, analyzed with an input rank for NMF of R = 4. From (a) to (c) we have: (a) the fiber proximal end image of 6 fluorescent bead fingerprint patterns overlapped on the sCMOS camera chip; (b) the short MMF located at a distance of 60 ± 10 µm from the fluorescent beads; (c) a CMOS Basler camera with the ground truth image of the sample. (d) The ground truth (GT) fingerprint patterns. (e) The fingerprint patterns obtained via NMF with rank 4. (f) The individual temporal activity traces of the sources obtained with NMF (blue) and their corresponding GT traces (gray). The red NMF trace (#4) is mainly background superimposed with the time trace activity of the other sources. (g) The Ground truth (GT) – NMF time trace correlations. The average diagonal value of the first 3 beads was $< \delta_{g,n} > = \delta_{avg}$ = 81.3% with standard deviation of σ_{δ} = 8.9%. (h) The GT-GT temporal trace correlations coefficients.

Figure S4: Results of the proof of principle experiment presented in Figure 2, analyzed with an input rank for NMF of R = 7.

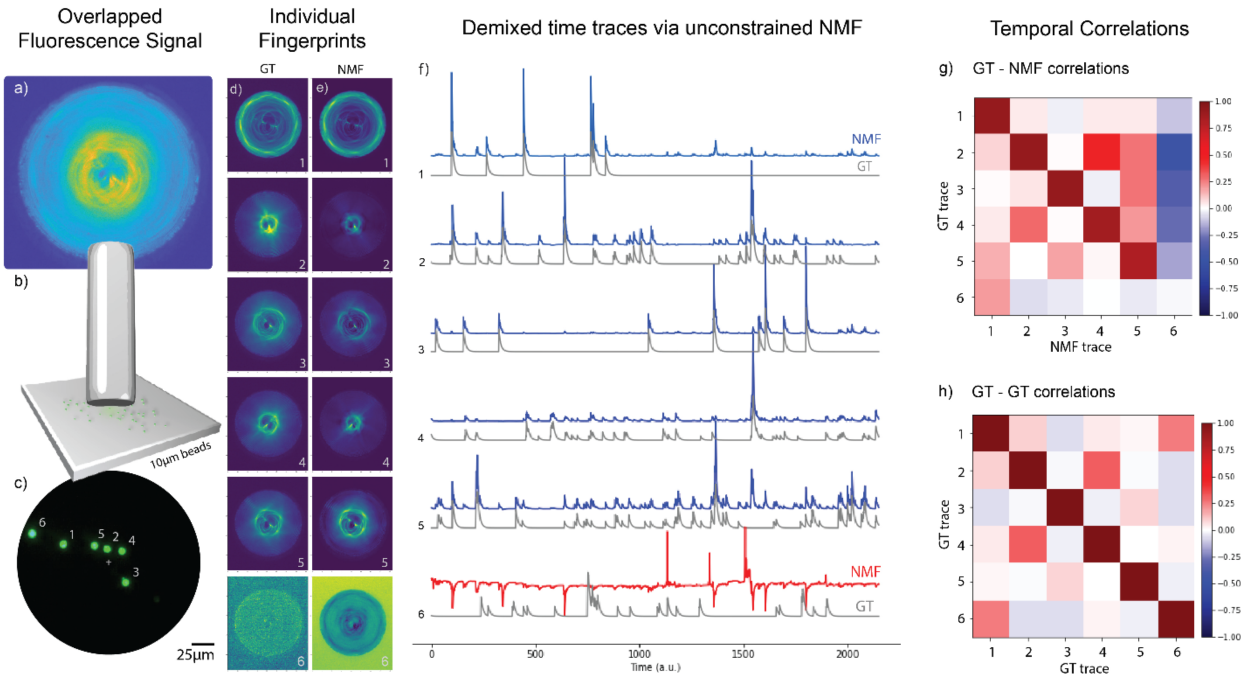

Figure S4 – Results of the proof of principle experiment presented in Figure 2, analyzed with an input rank for NMF of R = 7. From (a) to (c) we have: (a) the fiber proximal end image of 6 fluorescent bead fingerprint patterns overlapped on the sCMOS camera chip; (b) the short MMF located at a distance of 60 ± 10 µm from the fluorescent beads; (c) a CMOS Basler camera with the ground truth image of the sample. (d) The ground truth (GT) fingerprint patterns. (e) The fingerprint patterns obtained with the NMF. (f) The individual temporal activity traces of the sources obtained with NMF (blue) and their corresponding GT traces (gray). The red NMF trace (#6) was not recovered well by NMF since bead #6 was localized very close to the fiber core edge, therefore yielding low signal/contrast of its pattern (see GT scattering fingerprint of bead #6 in d). (g) The Ground truth (GT) – NMF time trace correlations. The average diagonal value of the first 5 beads was $< \delta_{g,n} > = \delta_{avg} = 88\%$ with standard deviation of $\sigma_\delta = 4\%$. (h) The GT-GT temporal trace correlation coefficients. The GT-GT correlation coefficients show that GTs from different sources were not fully uncorrelated, although each GT trace was unique in time (singular). For example, GT traces of beads #2 and #4 were fairly temporally correlated ($\gamma_{2,4} = \gamma_{4,2} = 31.2\%$, in (h)) and had a very clear spatial overlap (see GT and NMF scattering fingerprints #2 and #4 in (d) and (e)).

Figure S5: All the fingerprint results of the proof of principle experiment presented in Figure 2 (analyzed with NMF rank R = 9).

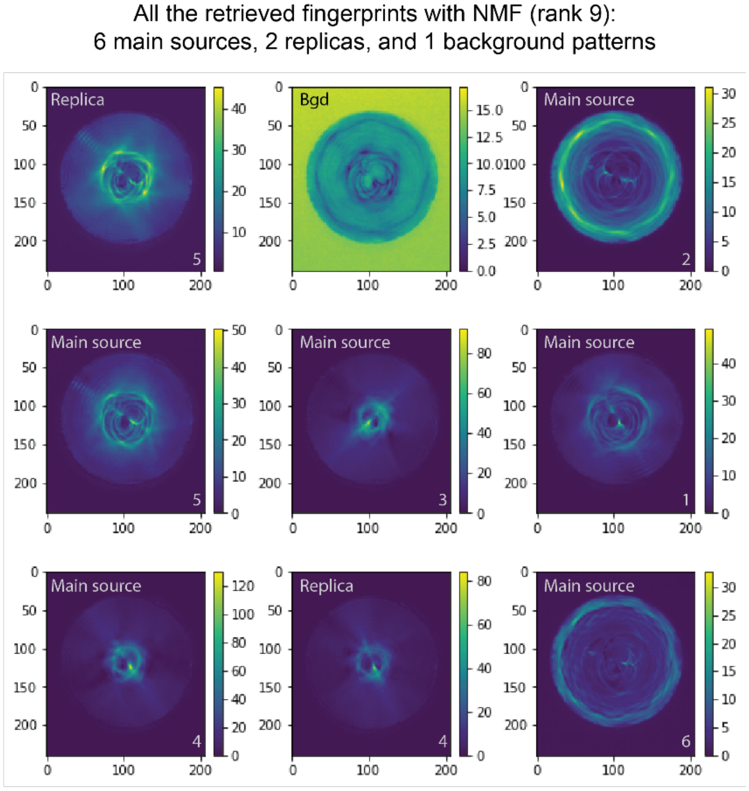

Figure S5 – All the fingerprint results of the proof of principle experiment presented in Figure 2 (analyzed with NMF rank R = 9). When rank is R = 9, the NMF demixes the data video in 6 main sources, 2 replicas, and 1 background (bgd). The number of the corresponding ground truth source is indicated in the bottom right corner of each fingerprint pattern.

Figure S6: NMF denoising effect on spatial fingerprints

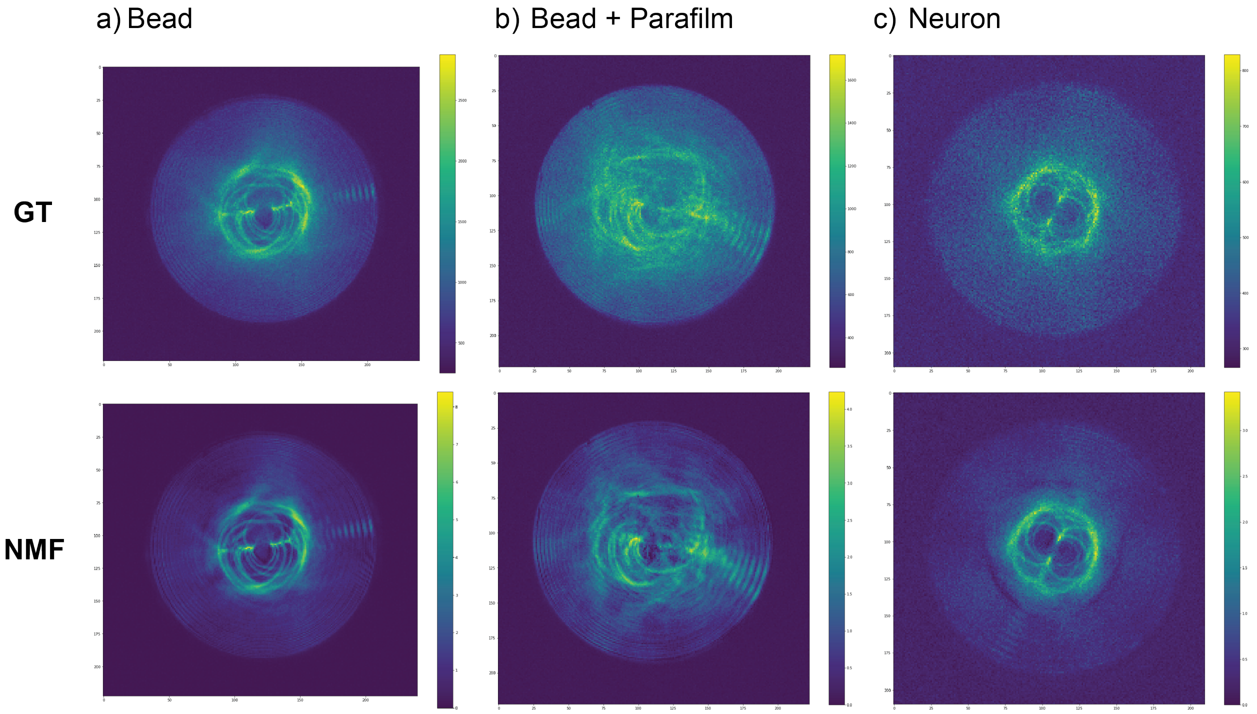

Figure S6 – NMF denoising effect on the retrieved spatial signal. Top: the ground-truth (GT) images. Bottom: the respective NMF fingerprint pattern. (a) Left column: GT-NMF patterns from bead #10 of Figure 3 (experiment without Parafilm M®). (b) Central column: GT-NMF patterns from bead# 09 of Figure 4 (experiment with Parafilm M®). (c) Right column: GT-NMF patterns of neuron #03 of Figure 6 from the main text (fixed brain slice experiment with Gad EGP labeled neurons). It is well-documented in the literature that NMF can denoise image data and we hypothesize that a long video recording can help NMF in denoising the pattern photometry data (Aonishi, T. et al. Neuroscience Research (2022); Varghese, K. et al. 3rd International Conference for Convergence in Technology (2018); Lin, B. et al. IEEE International Geoscience and Remote Sensing Symposium, (2018)). Importantly, NMF has already been used to simultaneously denoise, deconvolve and extract time traces from calcium imaging experiments (Pnevmatikakis, E.A. et al. Neuron (2016)).

Figure S7: Neuropil experiment results

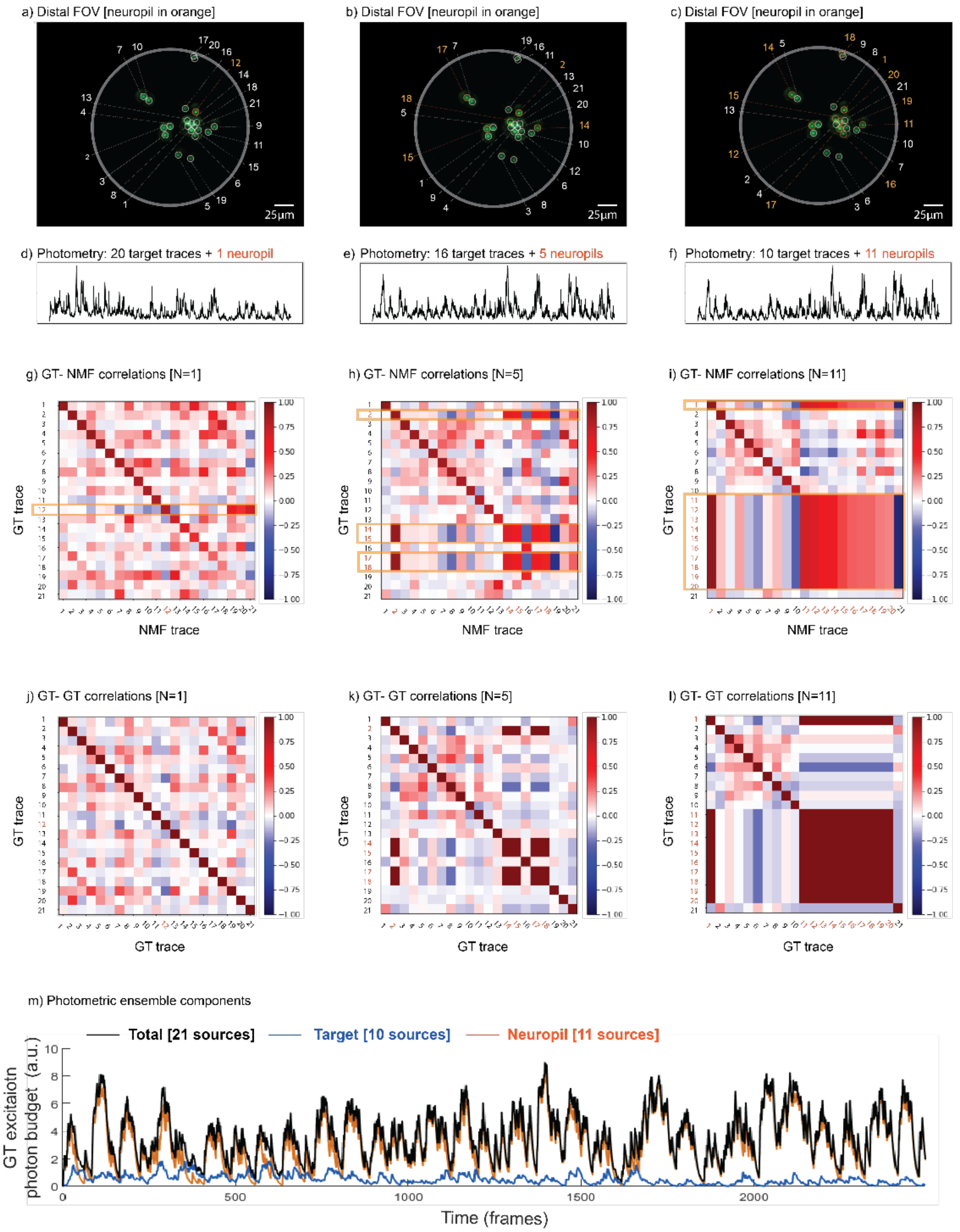

Figure S7 – Neuropil experiment results. NMF demixing with an increasing number of sources simulating neuropil signal. (a,b,c) The distal FOV with target sources (in white) and neuropil sources generate a dynamic background (in orange). The beads' indices are sorted from the highest to the lowest correlation between GT-NMF time traces. (d,e,f) Ensemble temporal signal (as in fiber photometry), (g,h,i) GT-NMF temporal correlations, (j,k,l) GT-GT temporal correlation of a proof-of-principle experiment designed with N=1, N=5, and N=11 beads (respectively) generating neuropil signal of out all the 21 sources. (m) Ground truth excitation photon budget of the experiment with N=11 neuropil sources over the video frames. In blue, the ensemble excitation time trace of 10 target sources added together; in red, the ensemble excitation time trace of the 11 neuropil sources; in black, the total ensemble excitation number of photons (sum of blue and red profiles). The average number of photons delivered to the neuropil sources (red trace mean value) was 86% of the average of the total number of photons delivered to all beads (black trace mean value).

Figure S8: Neuropil experiments estimated GT ranks: the fidelity plots

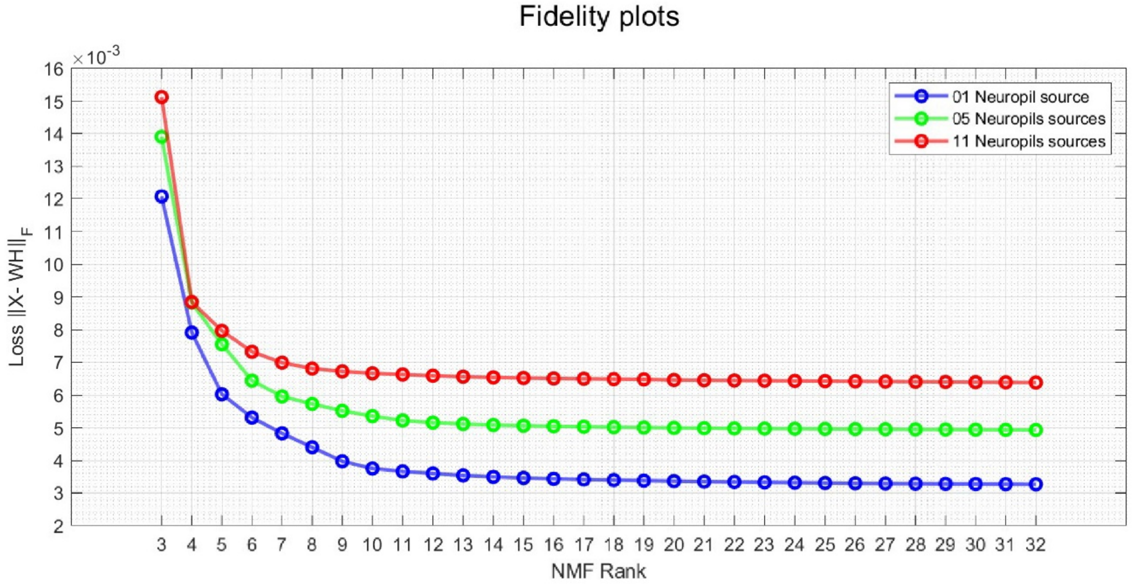

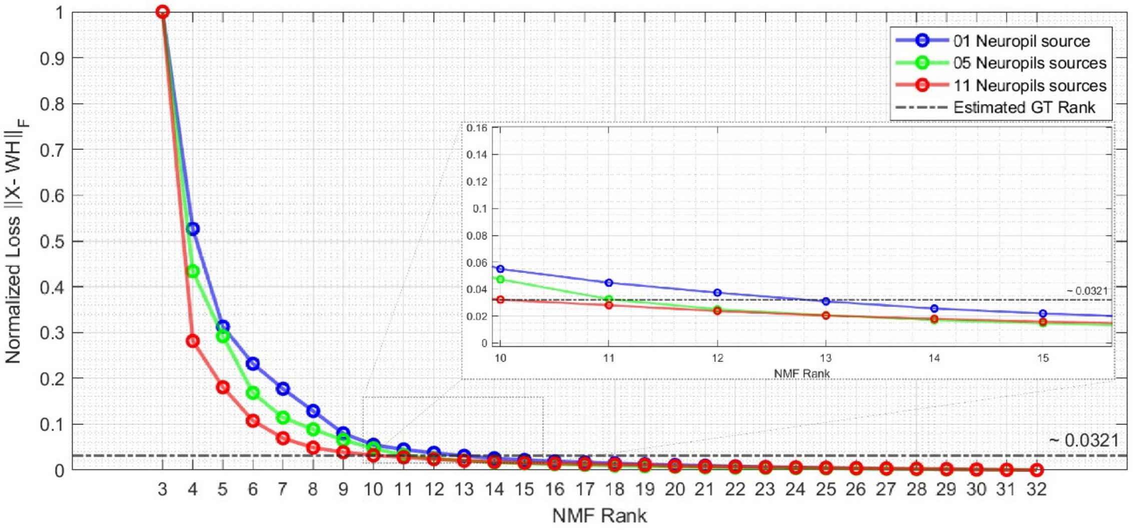

Figure S8 – Fidelity plots for different experiments performed with various number of neuropil sources, representing an estimation of NMF residual error as a function of the factorization rank (rank = 3, 4, 5, … , 32). Not-normalized (top) and normalized (bottom) residuals of 3 proof-of-principle experiments performed with 21 beads, including N=1 (blue), N=5 (green), N=11(red) neuropil sources, estimated with Frobenius norm (F) in the unconstrained NMF loss function. The inset in the normalized plot is the zoom of the highlighted rectangular region. The dashed-pointed horizontal line in the normalized plot (bottom), with value ~0.0321 taken as a common threshold in the normalized residuals over the 3 experiments (N=1, 5, and 11) (see below). The rank corresponding to this arbitrary threshold is a good estimation of the number of the well-retrieved target sources in the experiments. For example: 13 or 14 well-retrieved targets for N=1 (see also Figure S7.g), 11 or 12 targets for N=5 (see also Figure S7.h), and 10 of 11 targets for (N=11, see also Figure S7.i)). The value 0,0321 is the exact residual of the rank 10 in the red curve (N=11) and it was arbitrarily chosen to represent the threshold line. A subsequent and more detailed inspection of the patterns and individual time traces obtained with these estimated GT ranks would help to conclude the exact rank. For example, patterns with 2 clear defined rings are only generated by 2 sources at different radii, and time traces with non-realistic GECI profiles (baseline with negative peaks) should be disregarded in the analysis.

Figure S9: The miniscope detection sensitivity to a single source

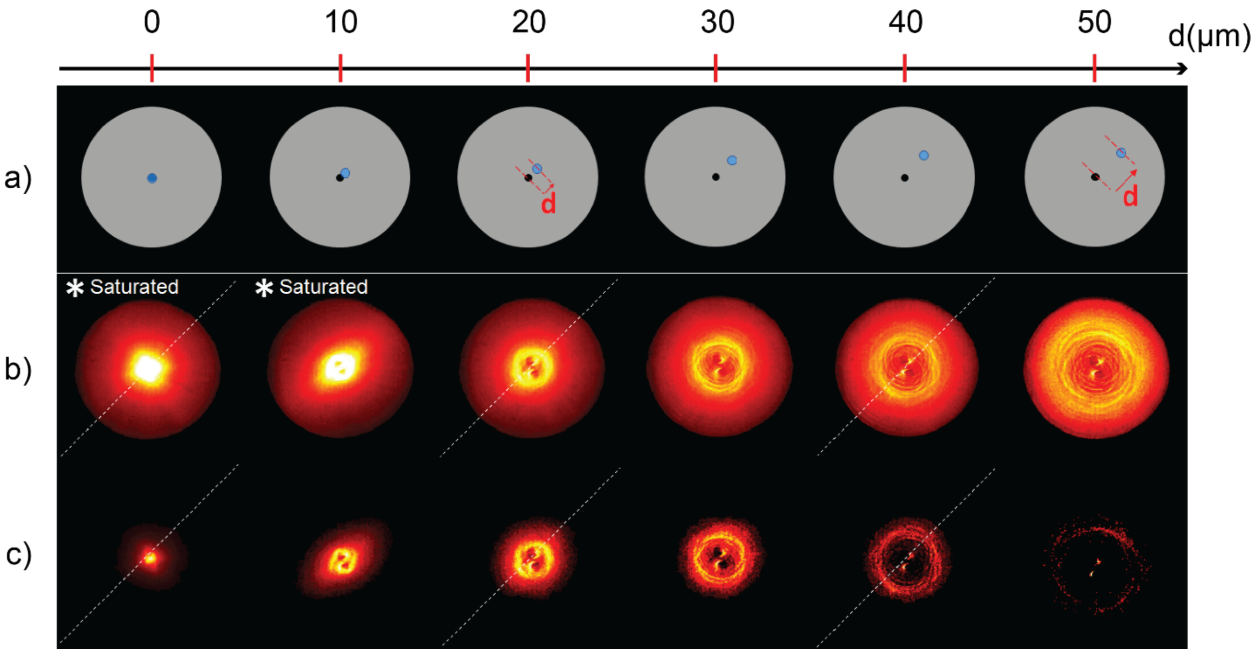

Figure S9 – Miniscope sensitivity to single-bead source upon different DAQ software setting conditions. (a) Illustration of the 10µm fluorescent bead displacement at the distal end of the fiber. The single-bead (blue dot) is displaced in a radial manner of a distance d (red vector) from the center of the fiber (black dot) generating different scattering fingerprints in (b) and (c). (b) Proximal end images of the fluorescence fingerprint patterns when the miniscope settings were for relatively low power and the slowest frame rate: LED power = 20%, corresponding to a transmitted power by the fiber of P_{MMF} = 9.49 ± 0.08 µW, and detection parameters of FPS = 10 Hz, Gain = 1.0. Under this condition, the detected patterns had saturated pixels (check the white pixels in the hot colormap) when the probed bead was closer to the axial center (d < 20µm). (c) Proximal end image of the detected fingerprint patterns when the miniscope settings were for very low power and the fastest frame rate: LED power = 10%, corresponding to a transmitted power by the fiber of P_{MMF} = 6.2 ± 1.2 µW, and detection parameters of FPS = 30 Hz, Gain = 1.0. The diagonal dashed line indicates the orientation of the single source displacement (at the fiber distal end) which coincides with the 2 bright spots orientation (see Supp info 04 for more details).

Figure S10: Pattern shape dependence on symmetrically positioned beads (with miniscope)

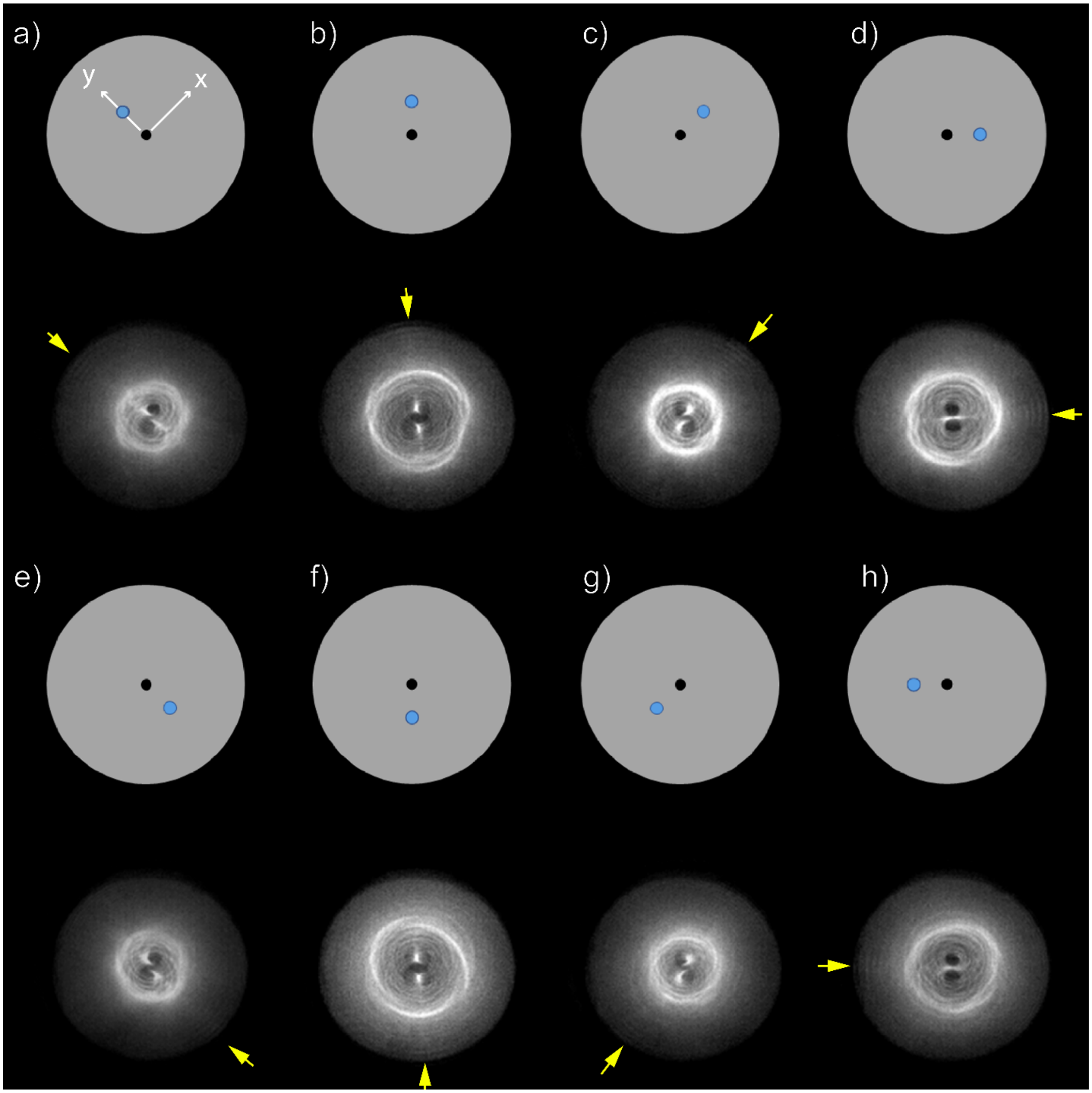

Figure S10 – Beads at different azimuthal orientational positions display rotated patterns in relation to each other. In (a) the white x and y arrows are the main axis of the miniscope optics. The flat gray circles on the top of the patterns illustrate the corresponding distal end of the fiber, where the blue dot represents the 10µm fluorescent bead, and the black dot represents the center of the fiber core. From (a) to (h), the axis pointing towards the bead is rotated by steps of 45°C. In each case, below each distal end cartoon, we show the corresponding proximal end data images (fluorescence patterns) obtained with the miniscope. Note that, the central structure of the fingerprint patterns (the core area within the bright ring with ramifications) contains bright and dark spots which rotate accordingly to the azimuthal orientation of the single source (blue dot) at the distal end. Yellow arrows point to some subtle bright features (ripples) at the patterns (d) and (h) that can possibly distinguish fingerprints generated from symmetrically positioned beads along one given direction if SNR is good enough. The very subtle shape differences between such two similar patterns from symmetric beads along one direction needs further investigation to confirm that they are really different, which is far from the scope of this work. In most cases, fingerprint pattern shapes seem to be different (not ambiguous) from beads localized at different orientations. Miniscope settings for these measurements were: LED = 30%, transmitted power was P_{MMF} = 13.1 ± 0.8 µW, and detection parameters of FPS = 20 Hz, Gain = 1.

Figure S11: Testing the results for NMF when spatially binning the recorded video

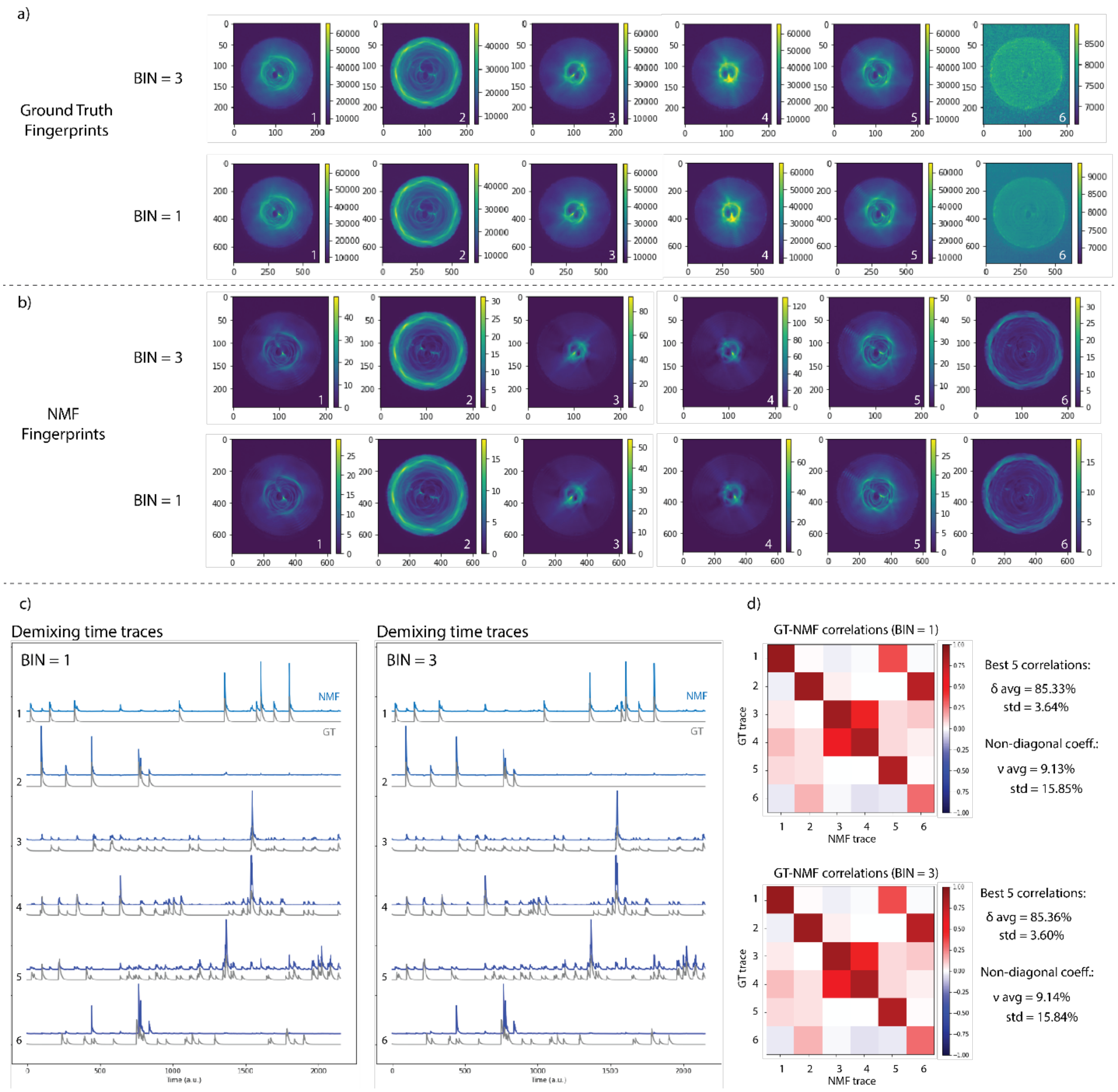

Figure S11 – Testing the results for NMF when spatially binning the recorded video, for the data also shown in Figure 2. The tested binning value was BIN = 3 (3x3 binning). (a) Ground truth fingerprint patterns with BIN = 3 (top row) and BIN = 1 (bottom row). (b) Fingerprint patterns extracted by NMF with BIN = 3 (top row) and BIN = 1 (bottom row). (c) GT time traces (grey) and NMF time traces (blue) for each bead with BIN = 1 (left) and BIN = 3 (right). (d). GT-NMF temporal correlation matrix obtained for BIN = 1 (top) and BIN = 3 (bottom). Overall results obtained with BIN = 3 are identical to results obtained with BIN = 1.

Supplementary Note 1: Estimating the number of fluorescence sources with NMF: the NMF rank study

The rank of NMF sets the number of components the NMF will demix from the video data (number of fingerprints and their corresponding time traces) [42-44,48]. In the experiment of Figure 2, we probed 6 beads in the FoV. In such case, because we know the number of sources, an expected good rank number ($R_{expected}$) as an input in NMF would be: $R_{expected} = 7$, because there were 6 beads + 1 background signal ($R_{expected} = N_{beads} + 1_{bgd}$). In a neuroscience experiment, one might not know *a priori* the number of the sources (neurons) one is probing, hence the input rank value on the NMF algorithm is not evident. However, one could still evaluate how well NMF performs to minimize the difference between the product of the two factorized spatial (W) and temporal (H) matrices and the original video matrix (X) with different rank values. The fidelity plot is shown in Figure S2. There, the residual error gradually decreases until reaching stabilized values, similar to a plateau (e.g., for rank values higher than 9). The curve profile changes more drastically its curvature around rank 4 and starts to stabilize around the expected rank $R_{expected} = 7$. Therefore, one could immediately make a rough estimation of the number of sources by just calculating the fidelity of NMF.

However, by investigating more closely these NMF results for ranks between 4 and 10, one might be able to count the number of individual sources with more precision. This is because the extra components are due to NMF forcing to split a single signal (fingerprint and time trace) into two or more positive signals. As a consequence, we expect to obtain fingerprint replicas of the bead patterns and background. For lower ranks, signal from individual sources can be superimposed with data from other sources or with the background. For example, in when we chose rank R =4, the background signal is still mixed with some of the fluorescent bead signal (see Figure S3). In such case, we retrieved relatively well the signal of 3 sources (there is some cross talk between bead 3 and the bead 4), but we lost the remaining ones.

For rank R = 7, we did retrieve well 5 unique fingerprints and their corresponding time traces, but we did not retrieve well the signal from bead #6 mostly due low SNR ratio. When using a higher rank and sorting the most correlated NMF time traces to the GTs, the background temporal correlations were higher than the time trace signal of the bead itself (see red time trace NMF curve in Figure S4). This result suggests that bead #6 was still embedded in the background for rank R=7.

For higher ranks than the expected one (R > 7), we start to split the signal of the source time traces, but also from the background (see replicas in Figure S5). For NMF rank = 9 (results shown in Figures 2 and S5) and beyond, NMF managed to find the hidden signal of bead #6 in the background component, which was correlating more with the GT of bead #6 than the background itself.

Supplementary Note 2: Comparing GT-NMF and GT-GT time trace correlation coefficients

The reason why we performed element-wise subtraction between GT-NMF and GT-GT correlation coefficients to evaluate the off-diagonal components is because the GT-GT time traces have some similarity among themselves, i.e., the non-diagonal GT-GT coefficients are not zero ($\nu_{i,j}^{GT-GT} \neq 0$). Consequently, it is expected that the non-diagonal elements of GT-NMF do not reach zero, but values close to the GT-GT coefficients. Let's consider the following notation for the time traces correlation coefficients:

- *GT-NMF time trace correlation coefficients*:

  GT-NMF diagonal coefficients = $\delta_{i,i}$

  GT-NMF non-diagonal coefficients = $\nu_{i,j}$

  For example, when the total number of sources N_s= 6:

$$\mathrm{R}_{\mathrm{GT-NMF}} = \begin{pmatrix} \delta_{1,1} & \nu_{1,2} & \nu_{1,3} & \nu_{1,4} & \nu_{1,5} & \nu_{1,6} \\ \nu_{2,1} & \delta_{2,2} & \nu_{2,3} & \nu_{2,4} & \nu_{2,5} & \nu_{2,6} \\ \nu_{3,1} & \nu_{3,2} & \delta_{3,3} & \nu_{3,4} & \nu_{3,5} & \nu_{3,6} \\ \nu_{4,1} & \nu_{4,2} & \nu_{4,3} & \delta_{4,4} & \nu_{4,5} & \nu_{4,6} \\ \nu_{5,1} & \nu_{5,2} & \nu_{5,3} & \nu_{5,4} & \delta_{5,5} & \nu_{5,6} \\ \nu_{6,1} & \nu_{6,2} & \nu_{6,3} & \nu_{6,4} & \nu_{6,5} & \delta_{6,6} \end{pmatrix} \quad (1)$$

- *GT-GT time trace correlation coefficients*:

  GT-GT non-diagonal coefficients = $\gamma_{i,j}$

  For example, when the total number of sources N_s= 6:

$$\mathrm{R}_{\mathrm{GT-GT}} = \begin{pmatrix} 1 & \gamma_{1,2} & \gamma_{1,3} & \gamma_{1,4} & \gamma_{1,5} & \gamma_{1,6} \\ \gamma_{2,1} & 1 & \gamma_{2,3} & \gamma_{2,4} & \gamma_{2,5} & \gamma_{2,6} \\ \gamma_{3,1} & \gamma_{3,2} & 1 & \gamma_{3,4} & \gamma_{3,5} & \gamma_{3,6} \\ \gamma_{4,1} & \gamma_{4,2} & \gamma_{4,3} & 1 & \gamma_{4,5} & \gamma_{4,6} \\ \gamma_{5,1} & \gamma_{5,2} & \gamma_{5,3} & \gamma_{5,4} & 1 & \gamma_{5,6} \\ \gamma_{6,1} & \gamma_{6,2} & \gamma_{6,3} & \gamma_{6,4} & \gamma_{6,5} & 1 \end{pmatrix}_{symmetric} \quad (2)$$

To better evaluate how well were the obtained time traces by the NMF, one should compute the absolute mean error between the non-diagonal elements of the GT-NMF and GT-GT values:

$$\zeta = \zeta_{i \neq j} = \begin{pmatrix} . & |\nu_{1,2}-\gamma_{1,2}| & |\nu_{1,3}-\gamma_{1,3}| & |\nu_{1,4}-\gamma_{1,4}| & |\nu_{1,5}-\gamma_{1,5}| & |\nu_{1,6}-\gamma_{1,6}| \\ |\nu_{2,1}-\gamma_{2,1}| & . & |\nu_{2,3}-\gamma_{2,3}| & |\nu_{2,4}-\gamma_{2,4}| & |\nu_{2,5}-\gamma_{2,5}| & |\nu_{2,6}-\gamma_{2,6}| \\ |\nu_{3,1}-\gamma_{3,1}| & |\nu_{3,2}-\gamma_{3,2}| & . & |\nu_{3,4}-\gamma_{3,4}| & |\nu_{3,5}-\gamma_{3,5}| & |\nu_{3,6}-\gamma_{3,6}| \\ |\nu_{4,1}-\gamma_{4,1}| & |\nu_{4,2}-\gamma_{4,2}| & |\nu_{4,3}-\gamma_{4,3}| & . & |\nu_{4,5}-\gamma_{4,5}| & |\nu_{4,6}-\gamma_{4,6}| \\ |\nu_{5,1}-\gamma_{5,1}| & |\nu_{5,2}-\gamma_{5,2}| & |\nu_{5,3}-\gamma_{5,3}| & |\nu_{5,4}-\gamma_{5,4}| & . & |\nu_{5,6}-\gamma_{5,6}| \\ |\nu_{6,1}-\gamma_{6,1}| & |\nu_{6,2}-\gamma_{6,2}| & |\nu_{6,3}-\gamma_{6,3}| & |\nu_{6,4}-\gamma_{6,4}| & |\nu_{6,5}-\gamma_{6,5}| & . \end{pmatrix} \quad (3)$$

So, the mean of all the non-diagonal values would be:

$$\zeta_{avg} = mean(\zeta_{i \neq j}) = \frac{1}{2\,(N_s - 1)} \left(\sum_{i \neq j}^{N_s - 1} |\boldsymbol{\nu}_{i,j} - \boldsymbol{\gamma}_{i,j}| \right) \quad (4)$$

And the standard deviation (std) of all non-diagonal elements would be:

$$\sigma_{\zeta} = std(\zeta_{i \neq j}) \quad (5)$$

Where N_s is the total number of sources. These two values ($\zeta_{avg} \pm \sigma_{\zeta}$) give us an estimation of the whole experiment quality since they should approach to zero.

Supplementary Note 3: Scattering properties of Parafilm M®

Parafilm M® is a well-known scattering media and it has similar scattering properties as the brain tissue [Boniface A. PhD dissertation (2020), Boniface A. Optica 2019]. One layer of Parafilm M® is approximately 120 µm thick, having a scattering mean free path of around $l_s \simeq 170$ µm and its transport mean free path close to $l_* \simeq 0.7$ µm, which leads to an anisotropy factor of $g \simeq 0.8$ for green light (532 nm). These values are comprehensively detailed in Antoine Boniface's PhD dissertation (2020) from our team and are very close to the values obtained in biological tissues, such as the brain tissue. Thus, one layer of Parafilm M® is assumed to possess scattering characteristics comparable to 120 µm of brain tissue.

Supplementary Note 4: Number of available scattering fingerprints (sources) to be demixed

We show in Figure 7 how patterns depend on the position of the source for a unique source. Patterns from 2 sources that are displaced by 10 µm along a radius of the fiber are already very different. In addition, we show that patterns of 2 sources that are localized with similar radial distances but different azimuthal angles around the fiber axis are also different (see patterns of beads 14 and 16 in Figure 3), and are well-demixed. In general, we show in Figure 3 that we can demix 10 µm diameter sources that are touching each other, indicating that their corresponding patterns are sufficiently different. Therefore, the number of available “scattering fingerprints” should be on the order of hundreds for fiber lengths of 5-15 mm whose fiber core has a similar area as the one we used. With this type of fiber, we show that we are already able to demix more than 20 sources, with and without a scattering layer between the sources and the fiber tip, because their spatial fingerprints are sufficiently different (Figures 3 and 4). Interestingly, however, we demonstrate that the presence of a scattering layer changes the spatial features of the fingerprint patterns, making them less similar to each other (less symmetric). This result suggests that sources at different depths in the brain can be demixed even if they overlap in x and y coordinates. In addition, this feature could facilitate our method in demixing time traces and suggests that it is possible to tune the total number of the probed sources if one decides to change the optical propagation properties of the system by engineering the implantable fiber. For example, one could add a scattering layer on the fiber distal end (the tip that faces the brain) by controlling its degree of polishing, or by simply choosing a fiber with different intrinsic propagation properties, such as NA, core geometry, etc. In theory, an infinitely long recording would be able to demix all the probed sources with unique spatial and temporal signatures. In practice, the maximum number of sources one can demix will depend on the unique features of the activity for each source, the signal-to-noise ratio, and the length of the recordings. Here, we use very short recordings (3000 frames), and the activity rate is the one observed in the mouse cortex.

Supplementary Information References

1. Aonishi, T. et al. Imaging data analysis using non-negative matrix factorization, Neuroscience Research (2022) https://doi.org/10.1016/j.neures.2021.12.001.
2. Varghese, K. et al. Denoising of Facial Images Using Non-Negative Matrix Factorization with Sparseness Constraint. 3rd International Conference for Convergence in Technology (2018) https://doi.org/10.1109/I2CT.2018.8529796.
3. Lin, B. et al. Hyperspectral Image Denoising via Nonnegative Matrix Factorization and Convolutional Neural Networks. IEEE International Geoscience and Remote Sensing Symposium, (2018) https://doi.org/10.1109/IGARSS.2018.8517388.
4. Pnevmatikakis, E.A. et al. Simultaneous Denoising, Deconvolution, and Demixing of Calcium Imaging Data. Neuron (2016). https://doi.org/10.1016/j.neuron.2015.11.037).
5. Boniface, A. Light control in scattering media and computational florescence imaging: towards microscopy deep inside biological tissues. (Laboratoire Kastler Brossel, L'Université Pierre et Marie Curie, 2020).
6. Boniface, A., Dong, J. & Gigan, S. Non-invasive focusing and imaging in scattering media with a fluorescence-based transmission matrix. *Nat Commun* **11**, (2020).
7. Gunaydin, L. A. *et al.* Natural neural projection dynamics underlying social behavior. *Cell* **157**, 1535–1551 (2014).
8. Kim, C. K. *et al.* Simultaneous fast measurement of circuit dynamics at multiple sites across the mammalian brain. *Nat Methods* **13**, 325–328 (2016).
9. Moretti, C. & Gigan, S. Readout of fluorescence functional signals through highly scattering tissue. *Nat Photonics* **14**, 361–364 (2020).
